# Microbial Mechanistic Insights into the Role of Sweet Potato Vine on Improving Health in Chinese Meishan Gilt Model

**DOI:** 10.3390/ani9090632

**Published:** 2019-08-30

**Authors:** Shengyu Xu, Pan Zhang, Meng Cao, Yanpeng Dong, Jian Li, Yan Lin, Lianqiang Che, Zhengfeng Fang, Bin Feng, Yong Zhuo, Jianping Wang, Zhihua Ren, De Wu

**Affiliations:** 1 Key Laboratory of Animal Disease-Resistant Nutrition, Ministry of Education; Key Laboratory of Animal Disease-Resistant Nutrition and Feed, Ministry of Agriculture and Rural Affairs; Key Laboratory of Animal Disease-Resistant Nutrition, Sichuan Province; Animal Nutrition Institute, Sichuan Agricultural University, Chengdu 611130, China; 2 Key Laboratory of Animal Disease and Human Health of Sichuan Province, Key Laboratory of Environmental Hazard and Human Health of Sichuan Province, College of Veterinary Medicine, Sichuan Agricultural University, Chengdu 611130, China

**Keywords:** Chinese Meishan gilt, sweet potato vine, endotoxin, microbial composition, intestinal health

## Abstract

**Simple Summary:**

Sweet potato vine as a source of fiber had been used in China for many years. We investigated the effects of fresh sweet potato vine on intestinal and plasma metabolites as well as colon microbial composition in Chinese Meishan gilts. Results suggest that sweet potato vine promoted intestinal muscle development, decreased gut permeability, endotoxin and pro-inflammatory cytokines concentrations, and increased butyrate production as well as beneficial flora, thus improving gut health.

**Abstract:**

This study explored the impact of fresh sweet potato vine on the growth as well as the metabolites and colon microbial composition in Chinese Meishan gilt. Twenty Meishan gilts (body weight 30 ± 0.18 kg, n = 10 per treatment) were randomly assigned to a control (CON) or sweet potato vine (SPV) supplementation diet treatment. Gilts were housed in individual stalls. In the SPV treatment, 2 kg fresh sweet potato vine was used instead of 0.18 kg basal diet which provided the same amount of digestive energy and crude protein with the exception of crude fiber (CON, 51.00 g/d vs. SPV, 73.94 g/d) in terms of dry matter intake. Gilts were slaughtered and samples were collected on day 19 after the third estrus cycle. The SPV treatment tended to increase slaughter weight of gilts (*p* = 0.07); it also increased (*p* < 0.05) gastrointestinal tract weight and intestinal muscle layer thickness. SPV treatment also decreased (*p* < 0.05) carcass yield and subcutaneous adipose tissue. The concentration of zonulin and endotoxin in plasma was decreased (*p* < 0.05) as the gilt consumed the SPV diet. Colonic fecal concentrations of endotoxin, lipocalin-2, and tumor necrosis factor-α (TNF-α) were decreased (*p* < 0.05), and interleukin-10 (IL-10) was increased (*p* < 0.05) in the SPV treatment. Butyric acid and acetate concentration in colonic content as well as acetate concentration in caecal content were increased (*p* < 0.05) in the SPV treatment. Furthermore, the expression of carnitine palmityl transferase (*CPT-1*) and peroxisome proliferator-activated receptor-α (*PPAR-α*) in gilt liver in SPV treatment was increased (*p* < 0.05) in comparison with CON treatment. Meanwhile, the composition of the colon microbes was also altered by SPV; representative changes included an increase in *Lactobacillus*, *Bacteroides*, *Roseburia*, and *Lachnospira.* These results indicate that gilt fed with sweet potato vine had decreased gut permeability, endotoxin and pro-inflammatory cytokines concentrations; colonic fecal microbiota was also changed, which may be further beneficial to the intestinal health of Chinese Meishan gilt.

## 1. Introduction

Sweet potato vine (SPV) is the by-product of sweet potato, consisting of leaf, stem, and stalk. In a previous study, we suggested that adding fresh SPV to gilt diet improved follicular development and thus benefited reproductive performance in Chinese Meishan (MS) gilt [1]. A large number of microorganisms are present in the intestine of pigs, which form a complex and relatively stable ecosystem with the body [2,3,4]. These microorganisms directly or indirectly participate in the nutrient metabolism and immune regulation of the host organism, but also suffer from various factors involving the immune system [2], intestinal environment [3], age [4], and diet [5]. Changes in the composition and metabolic activity of intestinal microbes caused by food intake have received extensive attention [5,6,7]. The SPV [dry matter, 10.13%; digestible energy (DE), kcal/kg, 2837.00; crude protein (CP), 14.90%, at a dry matter basis] contains crude fiber (CF, 15.51%), neutral detergent fiber (NDF, 21.63%), acid detergent fiber (ADF, 19.37%), and acid detergent lignin (ADL, 3.3%) [8,9]. However, the SPV as a source of fiber for remodeling microbiota in MS gilt has not yet been reported.

Dietary fiber have a significant effect on the microflora structure of pig intestines [10,11]. Concurrently, different fiber types and sources may directly affect the composition of intestinal colonies in monogastric animals [10,11]. Although different sources of fiber (maize fiber, soybean fiber, wheat bran fiber, and pea fiber) to feed fattening pigs were used, no difference in total bacteria populations was observed between diets. However, the composition of the microbial community was affected, in which lower ileal *Lactobacillus* populations and higher cecal *Escherichia coli* populations were found in a soybean fiber treatment in comparison with the control diet. Meanwhile, cecal *Lactobacillus* populations in pea fiber and *Bifidobacterium* populations in wheat bran were higher than control [10]. Feeding mice with soluble fiber (inulin) and insoluble fiber (cellulose) found that the addition of soluble fiber significantly increased the content of SCFA in the mouse cecum, indicating that soluble fiber is more easily fermented by intestinal microbes [11]. The addition of soluble fiber (inulin) increases the abundance of butyrate-producing bacteria (such as *E. rectale*, *Roseburia intestinalis*, and *Anaerostipes*) [12] while reducing the abundance of *Akkermansia muciniphila*, which affects the metabolism and absorption of host nutrients [13]. Zhou et al. (2017) [7] found that the addition of 1.5% soluble fiber inulin to the diet of a sow improved the maternal body weight, metabolism, inflammatory status, and distinct changes in the microbial community. The addition of SPV causes changes in the microbial fermentation substrate of the hindgut to affect the microbes in the intestines of the gilt. Whether SPV affects the gut health of the gilt by affecting the intestinal microbes and metabolites needs further study.

Therefore, this study investigated the effects of the sweet potato vine on the intestinal and plasma metabolites as well as the colon microbial composition in MS gilt. 

## 2. Materials and Methods

Animal procedures in this study were approved by the Animal Experimental Committee of Sichuan Agricultural University, under the Ethic Approval Code: SCAUAC201408-10.

### 2.1. Animals and Experimental Design

This study used 20 prepubertal MS gilts with weights at 30 ± 0.18 kg (109.00 ± 2.10 day). Gilts were randomly allocated to control (CON, n = 10) and sweet potato vine (SPV, n = 10) treatments. Gilts were housed in individual stalls, and each gilt was one replicate. The animals as well as their basal diets, feeding regime, and treatments were the same as those found in our previous study [1]. Briefly, basal diet included 3.08 Mcal DE /kg, 14.00% of CP, 0.68% Lysine, 0.53% calcium, and 0.48% phosphorus. Fresh SPV was provided as part (DE intake, 554 kcal/d; CP intake, 26 g/d,) of the basal diet to make sure gilt in SPV treatment had the same amount of energy and protein intake per day as in control group. CON treatment fed 1.64 kg basal diets per day. SPV treatment fed 1.46 kg basal diets and 2.00 kg fresh SPV per day. The two treatments offered a similar nutrient intake with the exception of CF (51.00 g/d vs. 73.94 g/d). Before feeding, fresh SPV was chopped into small pieces and then mixed with the powdered basal diet to feed the gilts. Gilts were fed twice daily (08:00 and 14:00) and water was offered ad libitum from a nipple drinker. The experiment ended at the morning of day 19 after the third estrus cycle of gilt.

### 2.2. Nutrient Digestibility Determination

When the body weight of gilt reached 40 kg, 12 healthy gilts (six gilts per treatment) were used in a nutrient digestibility trial to evaluate the effects of SPV on dry matter (DM), energy, nitrogen, CF, and fiber digestibility. Gilts were individually housed in metabolism cages and fed their respective experimental diets. The feeding was the same as the main study. The experimental diets were fed for a three-day adjustment period followed by a four-day formal feeding period. During the first and last day of formal feeding, 5 g ferric oxide was added to the basal diet as a color marker, respectively. Fecal samples were collected on a full day, on the first day at which red fecal samples appeared until the last day red fecal samples appeared. Samples were pooled, placed in plastic bags, and frozen at −20 °C. At the end of collection, feces were dried in a force-draft oven (65 °C) for 2 d and then weighed. Dried feces were ground through a 1-mm screen and frozen until subsequent fiber, nitrogen, crude fat, and gross energy determination could be performed. DM, ether extract (EE), and CF digestibility was determined using the AOAC procedure (2000) [14]. Nitrogen content of diets and feces was determined by the combustion method using a Leco TruSpec analyzer. Gross energy of feed and feces was determined by a Parr 6400 bomb calorimetry (Parr Instrument Co., Moline, IL, USA). 

### 2.3. Data Record and Sample Collection

On the morning of day 19 after the third estrus cycle of gilt (SPV treatment reached estrous 9.4 days later than control), live weight of all gilts (n = 10 / each treatment) was recorded, and then 10 mL of blood sample was collected by acute jugular puncture prior to slaughter to obtain the plasma. The plasma was stored at −20 °C until analysis. 

The gilts were slaughtered by an intracardial injection of sodium pentobarbital (50 mg/kg body weight) and bled by exsanguination. After the gilt was slaughtered, the head, tail, legs, and all internal organs (visceral organs and endocrine glands), as well as perirenal and omental adipose depots, were removed. The hot carcass weight was then recorded to determine dressing percentage. Loin muscle area was measured by planimetry on a cut at the level of the 13th and 14th ribs. Backfat thickness in carcasses represents the average of measures at the levels of the third and eleventh thoracic vertebrae and of the smallest measure over the gluteus medius muscle in the lumbar region according to Rehfeldt et al. (2012) [15]. For dissection-derived body composition, the left carcass was immediately further separated into muscle tissue, subcutaneous adipose tissue (SCAT), skin, and bones. 

After the stomach and entire intestine was rapidly removed, the weight of stomach, small intestine (include the duodenum, jejunum, and ileum), large intestine (include the cecum, colon, and rectum) was recorded. Three segments of 2 cm were cut at the mid-jejunum and mid-ileum separately, as described by Wang et al. (2014) [16]. The 2-cm intestinal segments were flushed gently with ice-cold PBS (pH 7.4) and then placed in 10% fresh, chilled formalin solution for histological measurement. A 10-cm section of caecum and colon was ligated and removed to avoid digesta admixture in different intestinal sections. The intestine section was placed on a chilled stainless steel tray. A small incision was opened in the distal end of each segment, and the digesta was collected in a 5-mL tube, then rapidly frozen in liquid nitrogen and stored at −80 °C. Samples of liver were collected, then quickly frozen in liquid nitrogen and stored at −80 °C until analysis.

### 2.4. Plasma Analyses

The concentrations of glucose (Glu), non-esterified fatty acid (NEFA), triglycerides (TG), total cholesterol (TC), and urea in plasma were detected by commercial kits according to the manufacturer’s instructions (Nanjing Jiancheng Bioengineering Institute, Nanjing, Jiangsu, China). Optical density (OD) values were determined at 520 nm, 440 nm, 546 nm, and 510 nm by a MuLtisKan MK3-Thermo Labsystems microplate reader (Thermo Labsystems, CA, USA). Plasma zonulin and endotoxin were measured using sow enzyme-linked immunosorbent assay (ELISA) Kits (R&D Systems Inc., Minneapolis, MN, USA). Optical density (OD) values were determined at 450 nm by a MuLtisKan MK3-Thermo Labsystems microplate reader (Thermo Labsystems, CA, USA). Minimal detection limits for Glu, NEFA, TG, TC, urea, endotoxin, and zonulin were 0.5 mmol/L, 10 μmol/L, 0.02 mmol/L, 0.1 mmol/L, 0.1 mmol/L, 3.1 ng/L, and 3 ng/L.

### 2.5. Analysis of Digesta

The endotoxin, lipocalin-2, interleukin-6 (IL-6), interleukin-10 (IL-10), tumor necrosis factor-α (TNF-α) of colonic digesta were measured using sow ELISA Kits (R&D Systems Inc., Minneapolis, MN, USA). The index analysis was performed following manufacturer’s instructions. Optical density (OD) values were determined at 450 nm through the use of a MuLtisKan MK3-Thermo Labsystems microplate reader (Thermo Labsystems, CA, USA). Minimal detection limits for endotoxin, lipocalin-2, IL-6, IL-10, TNF-α and zonulin were 3.1 ng/L, 10 ng/L, 12.5 ng/L, 5 ng/L, 7 ng/L, and 3 ng/L.

Short-chain fatty acids including acetate, propionate, and butyrate in digesta of caecum and colon were analyzed in a gas chromatographic system (GC, VARIAN CP-3800, Varian, Palo Alto, CA, USA). Briefly, a 2-g sample was suspended in distilled water (5 mL) and allowed to stand for 30 min, after which it was centrifuged at 4°C, 12,000 g for 10 min. The sample was then transferred to 2 mL of supernatant and mixed with 0.4 mL of metaphosphoric acid. Placed at 4 °C for 30 min, the sample was centrifuged again at 4 °C, 12000 g for 10 min before being transferred to 1.2 mL of supernatant and mixed with 15.2 μL of crotonic acid (210 mmol/L, internal standard), after which 0.3-mL liquid was mixed with 0.3-mL methanol. Finally, the mixed solution was filtered by the 0.22-μm filter membrane for the following gas chromatography analysis, which equipped with a micro-injector (10 μL), a flame ionization detector, and a capillary chromatographic column (CP-FFAP, 25 m × 0.32 mm × 0.3 μm).

### 2.6. Gene Expression

As described in our previous study [1], total RNA was extracted from frozen liver samples by TRIzol reagent (TaKaRa Biotechnology Co. Ltd., Dalian, China) according to the manufacturer’s instructions. Reverse transcription (RT) kit (Invitrogen, Carlsbad, CA, USA) was used to synthesize cDNA. Real-time PCR was used to quantify fatty acid synthetase (*FAS*), sterol regulatory element binding proteins (*SREBP-1*), carnitine palmityl transferase (*CPT-1*), and peroxisome proliferator activated receptor-α (*PPAR-α*) mRNA expression levels using the Sybr Green Kit (Qiagen, Valencia, CA, USA). Amplification was carried out with 12.5 µL final reaction volume containing 5 µL of Sybr green master mix, 0.5 µL of each primer (final concentration 0.25 µM), 6 µL of diethyl pyrocarbonate (DEPC)-water, and 0.5 µL of sample cDNA. All real-time PCRs were carried out in triplicate by a DNA Engine thermal cycler (PTC-0200, Chromo4 Real-Time detector, Bio-Rad, Hercules, CA, USA). The thermal cycling conditions were denaturation for 10 min at 95 °C, amplification for 45 cycles with denaturation at 95 °C for 20 s, annealing at 60 °C for 10 sec, extension at 72 °C for 9 sec, followed by fluorescence acquisition at 60 °C for 5 sec. Using the 2^−∆∆*C*t^ method to calculate the relative level of target gene expression [17], β-actin was amplified as housekeeping gene. Sequences of the primers are listed below. *FAS,* forward CTACATCGAGTGCATCAGACAGG and reverse GAACAGGAAGAGGCTGTGGTT; *SREBP-1*, TTGAGGACAGCCAGATGAAGC and reverse GCAGGAGAGACAGAGGAAGAC; *CPT-1*, CTACACAGAGACGGGGCACT and reverse AGGGCAAGAACTGGAAGCAG; *PPAR-α*, CTATCATTTGGTGCGGAGACC and reverse GGAGTTTGGGGAAGAGAAAGAC; *β-Actin*, forward GGCCGCACCACTGGCATTGTCAT and reverse AGGTCCAGACGCAGGATGGCG. 

### 2.7. Bacterial Community Analysis

The Mo Bio PowerFecal^TM^ DNA Isolation Kit (MO BIO Laboratories, Carlsbad, CA, USA) was utilized to extract the microbial DNA of thawed fecal sample (0.5 g). A nucleic acid/protein analyzer (Beckman DU-800, Beckman Coulter, Inc., CA, USA) was used to detect the concentration and purity of the genomic DNA. Following this, DNA samples were sent to perform amplicon pyrosequencing on Illumina HiSeq PE250 platforms at Novogene Bioinformatics Technology in Beijing, China. The V4 hypervariable region of the 16S rRNA gene was amplified using a forward primer 515f (5′-GTGCCAGCMGCCGCGGTAA-3′) and a reverse primer 806r (5′-GGACTACHVGGGTWTCTAAT-3′).

Operational taxonomic units (OTUs) come from high-quality tags utilizing Uparse v7.0.1001 (http://drive5.com/uparse/) at an identity threshold of 97% used Ribosomal Database Project (RDP) classifier Version 2.2 (http://github.com/rdpstaff/) to assign taxonomy for 16S rRNA gene sequences. The relative abundance of each OTU was explored at phylum and genus levels. A Venn diagram was generated for a comparison of the OTUs of the two treatments. Alpha diversity values for each sample were assessed by Qiime 1.7.0. (http://qiime.org/home_static/dataFiles.html) Principal coordinates analysis (PCoA) plots were produced using unweighted UniFrac metrics.

### 2.8. Statistical Analysis

**Figure 1:**
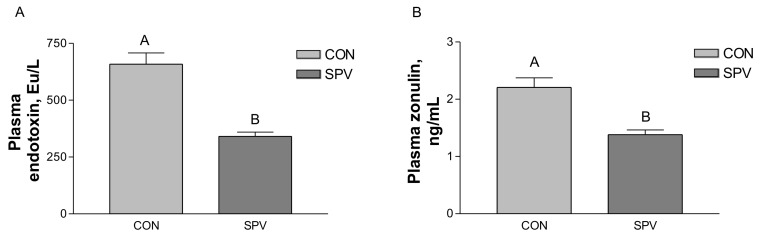
Effect of sweet potato vine treatment on plasma endotoxin (**A**) and zonulin (**B**) concentration in Meishan gilt. Values are mean ± SEM (n = 8). CON, gilts fed the basal diet; SPV, gilts fed basal diet with sweet potato vine. ^A, B^ Means not sharing identical superscripts in the same figure are significantly different (*p* < 0.01).

**Figure 2:**
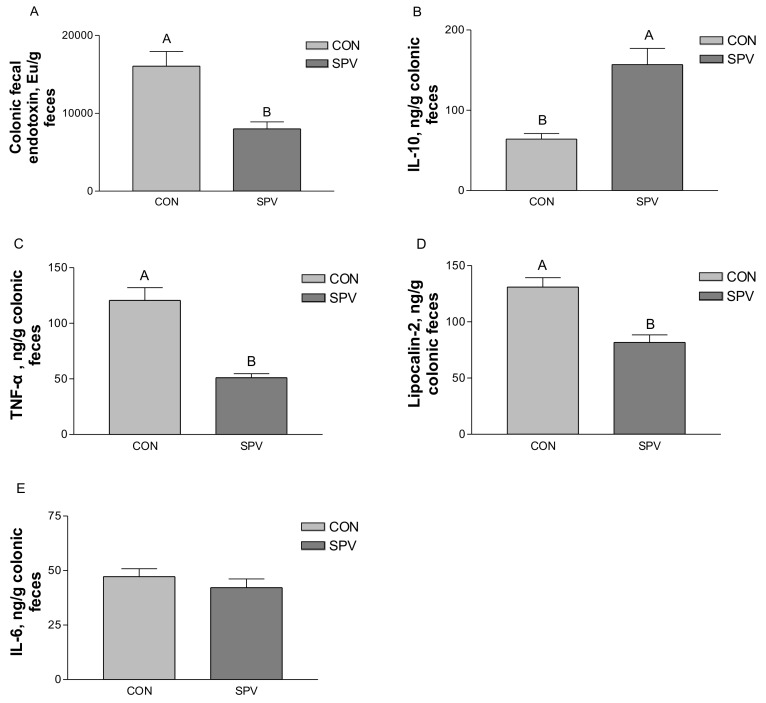
Effect of sweet potato vine supplementation on endotoxin (**A**), IL-10 (**B**), TNF-α (**C**), lipocalin-2 (**D**), and IL-6 (**E**) in colonic digesta of Meishan gilt. Values are mean ± SEM (n = 8). CON, gilts fed the basal diet; SPV, gilts fed basal diet with sweet potato vine. ^A, B^ Means not sharing identical superscripts in the same figure are significantly different (*p* < 0.01).

Data of relative abundance at phylum and genus level were log-transformed before statistical analysis. Following the normal distribution tested, data (Tables) were analyzed by an independent-samples t-test using SPSS 21.0 (IBM SPSS Company, Chicago, IL, USA). Other data, as seen in Figure 1 and Figure 2, were analyzed by t-test using GraphPad Prism analysis software. Differences were considered significant at *p* ≤ 0.05, whereas *p* < 0.10 was considered as a tendency. 

## 3. Results

### 3.1. Effect of Sweet Potato Vine on the Digestibility of Nutrients and Body Composition in Meishan Gilt

The results of nutrients’ digestibility trial have been shown in Table 1, and it can be observed that none of the DM, DE, CP, EE, and CF digestibility showed a significant difference between the two treatments (*p* > 0.05). Although no difference was detected on the dry matter intake between the two treatments, the SPV treatment had higher (*p* < 0.01) faecal dry matter output and lower faecal dry matter percentage (*p* < 0.01).

Body weight at slaughter of gilts in the SPV treatment tended to be heavier than those in the CON treatment (*p* = 0.07), as seen in Table 2. However, carcass weight and carcass length showed no significant difference between the two treatments. The stomach weight (*p* < 0.01), stomach index percentage (*p* < 0.01), small intestine weight (*p* < 0.01), large intestine weight (*p* < 0.01), jejunum muscle layer thickness (*p* < 0.05) and ileum muscle layer thickness (*p* < 0.01) was increased in the SPV treatment in comparison with the control. Results in body composition by manual dissection of the carcass showed that loin area (*p* < 0.05) and bone percentage (*p* < 0.05) were higher (*p* < 0.05), but backfat thickness tended to be thinner (*p* = 0.06), carcass yield, and subcutaneous adipose tissue (SCAT) was lower (*p* < 0.05) in the SPV treatment gilt in comparison with those in the CON treatment. Meanwhile lean meat and skin demonstrated no significant difference between the two treatments (*p* > 0.05).

### 3.2. Effect of Sweet Potato Vine on the Serum Index in Meishan Gilt

Meishan gilts fed with sweet potato vine had a higher concentration of glucose (*p* < 0.05, Table 3) and NEFA (*p* = 0.068) in the plasma than in the CON treatment. However, no difference was detected in the concentration of triglycerides, total cholesterol, and urea in the serum between the treatments. Meishan gilts in the SPV treatment had a lower (*p* < 0.05) plasma concentrations of endotoxin and zonulin than those in the CON treatment, as seen in Figure 1.

### 3.3. Effect of Sweet Potato Vine on the Inflammation State of the Gilts’ Intestinal Mucosal Epithelium in Meishan Gilt

Several biomarkers of inflammation were measured to reflect the effects of SPV on the inflammatory response in gilts’ intestines. As shown in Figure 2, concentrations of endotoxin, lipocalin-2, and TNF-α (pro-inflammatory cytokines) in colonic digesta were decreased, and IL-10 (anti-inflammatory cytokines) was increased in SPV treatment in comparison with the CON treatment. However, no significant difference was found in colonic digesta IL-6 between the treatments. This suggests that Meishan gilt fed SPV showed a low-grade inflammation of mucosal surfaces in the intestine.

### 3.4. Effect of Sweet Potato Vine on the SCFA Concentrations in Meishan Gilt

As shown in Table 4, butyric acid concentration in colonic content (*p* < 0.05), acetate concentration in caecal content (*p* < 0.05), and colonic content (*p* < 0.05) were both significantly increased; concurrently, butyric acid concentration in caecal content tended (*p* = 0.07) to be increased in the SPV treatment in comparison with those in the CON treatment. But propionic acid concentration either in caecal content or in colonic content did not differ (*p* > 0.05) between the two treatments.

### 3.5. Effect of Sweet Potato Vine on the Fatty Acid Synthesis and Catabolism Related Gene Expression in Meishan Gilts

**Figure 3:**
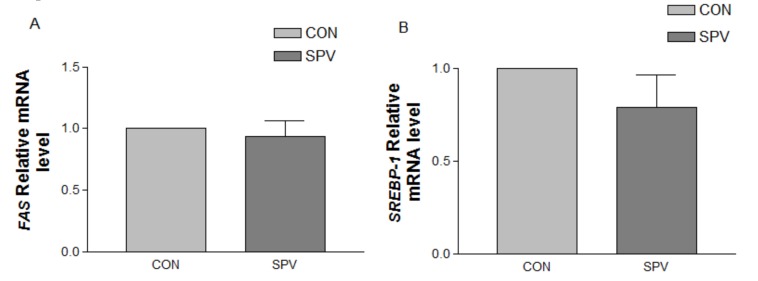

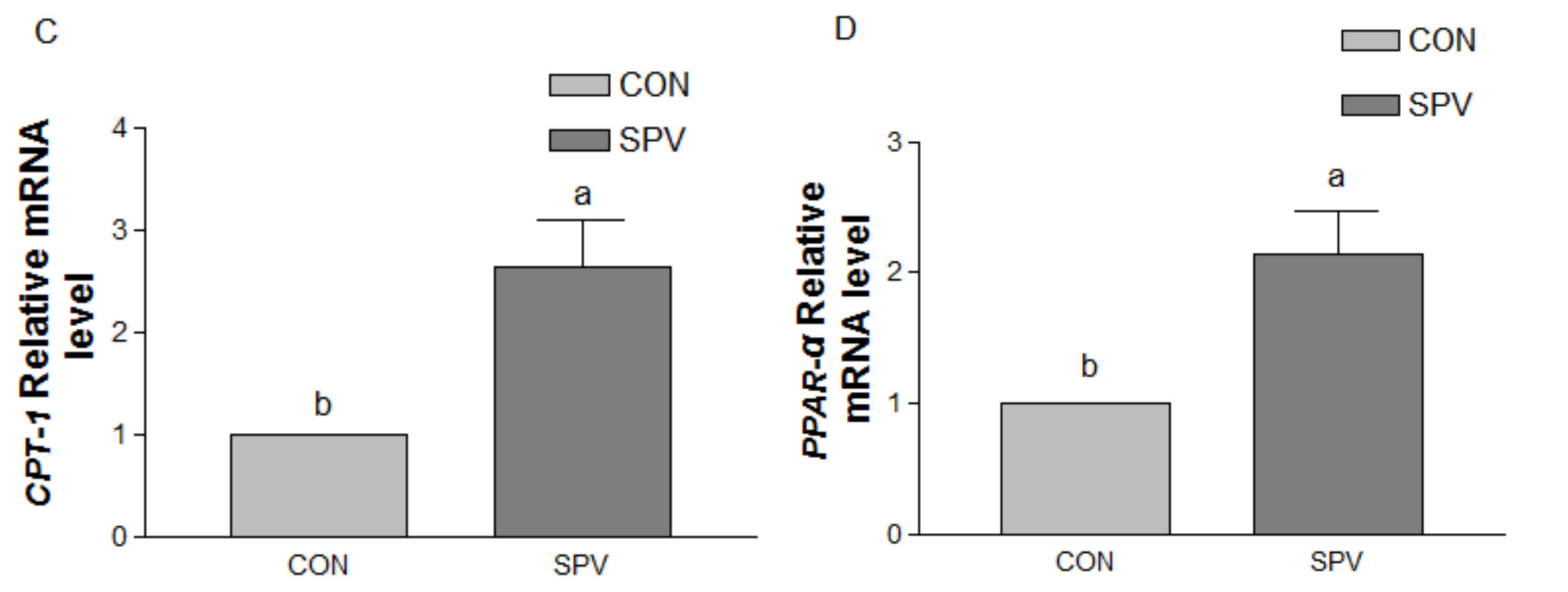
Effect of sweet potato vine treatment on *FAS* (**A**), *SREBP-1* (**B**), *CPT-1* (**C**), and *PPAR-α* (**D**) genes’ mRNA expression in liver of Meishan gilt. Values are mean ± SEM (n = 8). CON, gilts fed the basal diet; SPV, gilts fed basal diet with sweet potato vine; *FAS*, fatty acid synthetase; *SREBP-1*, sterol regulatory element binding proteins; *CPT-1*, carnitine palmityl transferase; *PPAR-α*, Peroxisome proliferator activated receptor-α. ^a, b^ Means not sharing identical superscripts in the same figure are significantly different (*p* < 0.05).

We also evaluated mRNA expression levels of fatty acid synthesis and catabolism in liver, as seen in Figure 3. There was no treatment effect on the fatty acid synthesis related gene *FAS* and *SREBP-1* mRNA expression, as seen in Figure 3A,B. However, Meishan gilt fed sweet potato vine had increased (*p* < 0.05), as seen in Figure 3C,D, gene expression level of *CPT-1* and *PPAR-α* in the liver in comparison with control. 

### 3.6. Effect of Sweet Potato Vine on the Microbiota of the Colon in Meishan Gilt

**Figure 4:**
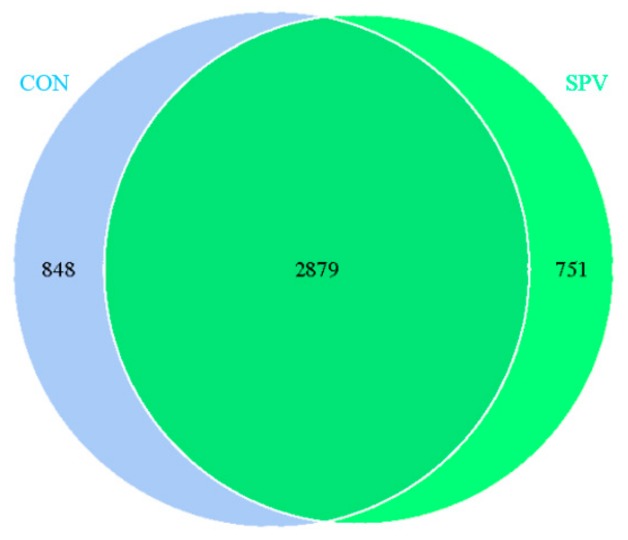
Microbiota comparison of the OTUs between the treatments in colon. CON, gilts feed the basal diet; SPV, gilts fed basal diet with sweet potato vine.

**Figure 5:**
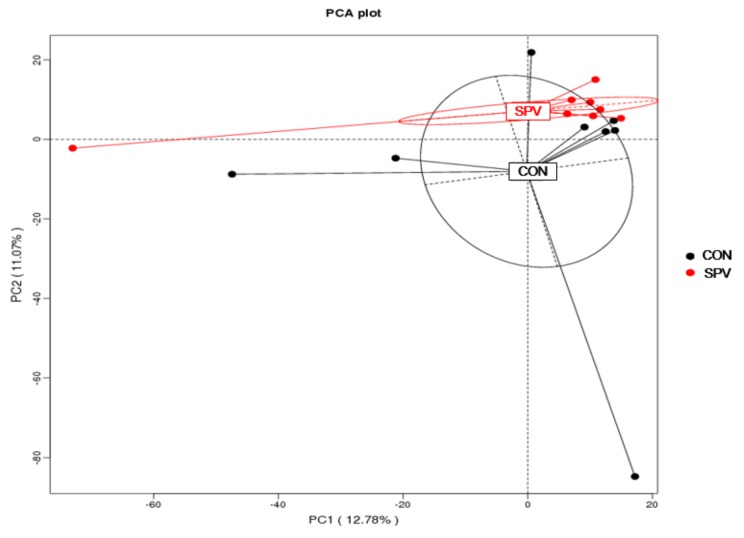
Comparison of the colon microbiota composition between the treatments. Principal component analysis to visualize the unweighted UniFrac distances of colon digesta samples from individual gilt. CON, gilts fed the basal diet; SPV, gilts fed basal diet with sweet potato vine.

Tags and OTUs number for the two treatments are shown in Table 5. As seen in Figure 4, 2879 OTUs existed in the two treatments and were called core OTUs. In comparison, 848 and 751 OTUs were uniquely identified at CON and SPV, respectively. There was no significant difference in the Shannon index, observed species and Chao index from the alpha diversity analysis. For beta diversity analysis, we examined the relationship in colon microbiome between the CON and SPV using the Principal Component Analysis (PCA). The distribution of microbiota of CON and SPV was distinctly clustered separately, as seen in Figure 5, indicating that SPV significantly affected the colon bacterial structure.

**Figure 6:**
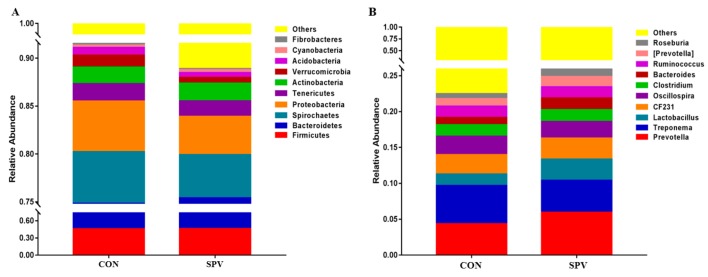
16S rRNA gene analysis reveals phyla (**A**) and genus (**B**) level differences between the treatments. CON, gilts feed the basal diet; SPV, gilts fed basal diet with sweet potato vine.

The effects of SPV on the relative abundance at phyla and genus level of the colon microbiota are displayed in Figure 6. The top six dominated phyla are *Firmicutes*, *Bacteroidetes*, *Spirochaetes*, *Proteobacteria*, *Tenericutes*, and *Actinobacteria*, as seen in Figure 6A. The abundant phyla are *Firmicutes* and *Bacteroidetes* which accounted for more than 75%. The relative abundances of the top ten dominated genera are presented in Figure 6B. Furthermore, the phyla (>0.01 %) and genera (>0.3 %) were chosen for significance analyses. The SPV treatment decreased the relative abundance of *Verrucomicrobia* (*p* < 0.05), as seen in Table 6, *Euryarchaeota* (*p* = 0.09) and *Lentisphaerae* (*p* = 0.09) at phyla level. At genera level, *Lactobacillus*, *Bacteroides*, *Roseburia* and *Lachnospira* were significantly higher (*p* < 0.05), but *SMB53* was lower (*p* < 0.05) in the SPV treatment than that in CON, as seen in Table 7.

## 4. Discussion

This was the first study to focus on the effect of SPV on intestinal digestion, the intestinal and plasma metabolites, and colon microbial composition in Chinese Meishan gilts. In this study, the addition of fresh SPV did not significantly affect the apparent digestibility of nutrients. On the basis of our original knowledge, the fiber in the diet cannot be digested in the small intestine; it will adhere and dilute other nutrients, thereby interfering with the absorption of other nutrients. Accordingly, the addition of fiber may not be conducive to production. Previous studies have confirmed that increased fiber levels in the diet are not beneficial to the digestion of nutrients, such as crude protein, energy, and fat [18,19]. After the addition of four different levels of fiber into the diets of four different hybrid pig breeds, the results showed that dietary fiber levels significantly affected the apparent digestibility of nutrients. With the reduction of local pig bloodline, the digestibility of acidic and neutral detergent fibers gradually decreased [20]. However, the use of fresh alfalfa instead of a part of basal diet to feed the finishing pigs showed that the apparent digestibility of dry matter, crude protein, and crude ash increased compared with the control group, but the digestibility of crude fat tended to decline [21]. These results suggest that the apparent digestibility of nutrients in pig diets varies according to the fiber amount and type, and the local Chinese pig breed exhibits much more fiber tolerance. The MS gilt may be tolerant of higher fiber levels. In this study, we used fresh SPV, which had lower dry matter, and may not have reached an extent to which apparent digestibility was substantially affected. In addition, this experiment utilized the principle of equal energy and nitrogen substitution; the two treatment gilts had almost the same dry matter intake. 

In the current study, the relative weight of the stomach, small intestine, and large intestine of MS gilt, as well as the thickness of the muscle layer of jejunum and ileum was increased significantly in SPV treatment. The addition of fiber into the diet affects the development of the gut of pigs [22,23]. Using high-, medium- and low-fiber diets to feed the piglets, the results demonstrated that a high fiber addition promoted the development of stomach and duodenum and increased the index of jejunum, ileum, cecum, and colon [22]. An increase in the amount of NDF in the diet of piglets also resulted in an increase in weight and length of the large intestine [23]. This might be attributable to the fiber in fresh SPV enters the digestive tract after which it swells and compresses gastrointestinal tract of the gilts, causing the weight and length of gastrointestinal tract to increase, resulting in a decrease in the slaughter rate (67.51% versus 63.68%) in this study. Moreover, the results of the body composition by manual dissection of the carcass in the present study showed that gilts in the SPV treatment had thinner backfat thickness and less percentage of subcutaneous adipose tissue; such evidence indicates that SPV could slow fat deposits. Consistent with these results, we also found that SPV treatment increased non-esterified fatty acid in the plasma as well as fatty acid catabolism gene expression level of CPT-1 and PPAR-α in the liver. Such results support our previous research that gilt in the SPV treatment reached puberty 9.4 days later than those in the CON treatment [1]. Previous studies have proposed that puberty occurs only after the attainment of a minimum level of leanness or fatness, or ratio of fat to lean [24]. Thus, gilt in the SPV treatment spent much more time to reach the minimum level of fatness to trigger the onset of puberty in comparison with those in the CON treatment.

In this study, the concentrations of butyric acid and acetate in caecal content and colonic content of gilt in SPV treatment were both increased; however, the concentrations of propionic acid did not. Supplemental fiber with different fiber components resulted in inconsistent SCFA production in the intestinal tract of pigs [25]. Our results might be closely related to the fiber monosaccharide composition [26]. Fibers with a high uronic acid content increased acetic acid production, fibers with high glucose content increased propionic acid production, and xylose promoted the production of butyric acid [27]. Therefore, the content of uronic acid and xylose in the fresh SPV was relatively high; accordingly, the acetic acid and butyric acid produced by the fermentation were significantly enhanced. Studies in both rats and humans have demonstrated that SCFA can decrease the concentration of cholesterol in the blood [28,29], thus affecting lipid metabolism. However, in this study, SPV did not significantly reduce triglycerides and total cholesterol in the MS gilt blood. In addition, several studies have demonstrated that SCFA activates the oxidation of fatty acids by activating activity of AMPK in liver and muscle cells, inhibiting the denovo biogenesis of fatty acids and lipid catabolism [30]. Accordingly, this may explain the slower fat deposition of gilt in the SPV treatment. 

Zonulin concentration may enhance in blood when intestinal mucosal integrity is impaired [31]. A previous study has found that, via a modulation of expression of tight junction proteins, butyrate maintains an intestinal barrier integrity [32]. In this study, we observed that gilt fed SPV diets experienced decreased levels of zonulin in plasma and an increase in butyric acid concentration. Furthermore, SPV treatment reduced endotoxin in blood and colonic feces. In the colonic feces analysis, the anti-inflammatory cytokines IL-10 increased, and pro-inflammatory cytokines TNF-α and lipocalin-2 decreased in the SPV treatment. Cytokines play an important role in the inflammatory response [33]. From these results, it may be concluded that SPV treatment decreased the gut permeability, ameliorated the inflammation state of intestinal mucosal epithelium as indicated by decreasing the zonulin concentration, increased the butyrate, reduced endotoxin, and changed inflammatory cytokines. 

Abundant phyla *Firmicutes* and *Bacteroidetes* could be found in more than 75% of the colonic feces in this study, which play a role in degrading polysaccharides and promoting energy absorption in the gilt intestinal tract. Fresh SPV contained cellulose, hemicellulose, and pectin, which provided the substrate for the intestinal fiber-degrading bacteria. The microbes in the intestines of animals are “organs” that are composed of a series of microorganisms such as bacteria, archaea, and fungi. Microbes form a symbiotic relationship with the animal body and maintain the homeostasis of the intestinal environment. The hindgut digestion of a pig is mainly microbial digestion, in which the colon is the most developed in the hindgut. Most of the hard-to-digest fiber materials are decomposed by microbial fermentation in the hindgut to produce products such as acetic acid and butyric acid. The proportion of *Firmicutes* and *Bacteroidetes* is related to the body’s energy metabolism [34]. In this study, the SPV feed increased the abundance of butyrate-producing bacteria like *Roseburia* and *Lachnospira* in the colonic feces in Meishan gilts. *Roseburia* was part of commensal bacteria producing short-chain fatty acids, especially butyrate, affecting the colonic motility, immunity maintenance, and anti-inflammatory properties [35]. It also reported that the reduction in *Roseburia* contributed to the dysbiosis of ulcerative colitis patients [36]. Weber [37] and other studies have found that the *Lachnospira* is the main pectin degrading bacterium in the human intestine. At the same time, *Bacteroides*, *Lactobacillus*, and *Bifidobacteria* can grow in the presence of pectin and pectin oligosaccharides [38]; pectin contained in the SPV may provide conditions for the growth of these bacteria. Accordingly, the bacterial abundance such as *Lactobacillus* and *Bacteroides* was enhanced in this study. Therefore, the SPV feed provided different fermentation substrates for the fermentation of intestinal microorganisms, thereby affecting the abundance of the bacteria and the SCFA production.

On the other hand, beneficial microorganisms colonized the intestinal mucosa to constitute a protective barrier to prevent colonization by other pathogenic bacteria [39]. Pathogenic bacteria in the intestinal tract are harmful to the mucosal barrier and may induce intestinal diseases. In this study, SPV treatment increased the abundance of *Lactobacillus* and *Bacteroides*. *Lactobacillus* is the only genus of the *Lactobacillus* family, including the species of *Lactobacillus mucosae* and *Lactobacillus reuteri*. *Lactobacillus* presents in the gut of animals and plays a major role in maintaining bacterial balance and preventing colonization of pathogenic microorganisms. *Lactobacillus* enters the intestine after being adhered to the intestinal mucosa, during which a biological barrier is formed that can prevent the invasion of pathogenic microorganisms [40,41]. Simultaneously, *Lactobacillus reuteri* can also secrete reuterin, which has a strong inhibitory effect on harmful bacteria [42]. Therefore, SPV treatment may improve the intestinal health by promoting the abundance of certain beneficial bacteria, thereby inhibiting the growth of pathogenic bacteria.

## 5. Conclusions

In conclusion, feeding fresh sweet potato vine instead of a part of basal diet with the same amount of energy and protein to Chinese Meishan gilts might promote the intestinal development and improve gut health, indicated by the prevented gut permeability, decreased endotoxin, and inflammation of mucosal surfaces, as well as increased SCFA production and beneficial flora.

## Figures and Tables

**Table 1:** The digestibility in Meishan gilts in different treatments.

Item	CON	SPV	*p*-Value
DMI, kg/d	1.44 ± 0.01	1.48 ± 0.02	0.95
FDMO, kg/d	0.80 ± 0.08	0.97 ± 0.03	<0.01
FDM, %	35.16 ± 1.97	30.54 ± 1.10	<0.01
DM, %	80.1 ± 0.5	80.1 ± 0.4	0.92
DE, %	79.1 ± 0.5	78.2 ± 0.4	0.21
CP, %	80.5 ± 1.0	79.7 ± 1.2	0.62
EE, %	69.6 ± 3.3	65.7 ± 2.9	0.39
CF, %	39.6 ± 1.3	36. 6 ± 2.0	0.23

CON, gilts fed the basal diet; SPV, gilts fed basal diet with sweet potato vine. Values are mean ± SEM (n = 6). DMI, dry matter intake; FDMO, faecal dry matter output; FDM, faecal dry matter percentage; DM, dry matter; DE, digestible energy; CP, crude protein; EE, ether extract; CF, crude fiber.

**Table 2:** Effect of the sweet potato vine treatment on visceral weight and body composition by manual dissection of the carcass in Meishan gilts.

Item	CON	SPV	*p*-Value
Body weight, kg	62.20 ± 2.20	70.20 ± 3.40	0.07
Stomach, kg	0.53 ± 0.03	0.71 ± 0.04	<0.01
Stomach index, %	0.80 ± 0.03	0.94 ± 0.03	<0.01
SI weight, kg	1.11 ± 0.03	1.42 ± 0.03	<0.01
SI index, %	1.81 ± 0.09	2.08 ± 0.12	0.12
Jejunum muscle layer thickness, μm	678.50 ± 37.70	1003.50 ± 94.70	0.01
Ileum muscle layer thickness, μm	889.80 ± 35.00	1229.80 ± 71.80	<0.01
LI weight, kg	1.24 ± 0.06	1.48 ± 0.04	<0.01
LI index, %	2.08 ± 0.06	2.10 ± 0.11	0.91
Carcass weight, kg	42.04 ± 2.35	46.18 ± 3.30	0.33
Carcass length, cm	84.40 ± 1.70	88.20 ± 3.50	0.37
Loin area, cm^2^	19.83 ± 1.27	24.10 ± 1.30	0.03
Backfat thickness, mm	15.15 ± 1.25	12.14 ± 0.83	0.06
Carcass yield, %	67.51 ± 1.09	63.68 ± 1.22	0.04
Carcass tissues			
SCAT, %	28.92 ± 1.50	24.95 ± 0.85	0.03
Lean meat, %	43.07 ± 1.00	44.96 ± 0.58	0.12
Bone, %	10.24 ± 0.18	11.29 ± 0.34	0.02
Skin, %	17.76 ± 0.58	18.79 ± 0.67	0.27

CON, gilts fed the basal diet; SPV, gilts fed basal diet with sweet potato vine. Values are mean ± SEM (n = 10). SI, small intestine, include the duodenum, jejunum and ileum; LI, large intestine, include the cecum, colon and rectum; SCAT, subcutaneous adipose tissue. Related to left-half carcass.

**Table 3:** Effect of sweet potato vine treatment on plasma metabolic substrates in Meishan gilts.

Item	CON	SPV	*p*-Value
Glu (mmol/L)	3.69 ± 0.11	5.32 ± 0.25	0.000
NEFA (μmol/L)	68.8 ± 11.7	104.9 ± 14.0	0.068
TG (mmol/L)	0.21 ± 0.02	0.22 ± 0.02	0.469
TC (mmol/L)	2.09 ± 0.11	1.99 ± 0.16	0.645
Urea (mmol/L)	3.96 ± 0.28	4.23 ± 0.32	0.541

CON, gilts feed the basal diet; SPV, gilts fed basal diet with sweet potato vine. Values are mean ± SEM (n = 8). Glu, glucose; NEFA, Non-esterified fatty acid; TG, Triglycerides; TC, Total cholesterol.

**Table 4:** Effect of sweet potato vine treatment on concentrations of acetate, propionic acid, and butyric acid in caecal and colonic digesta of Meishan gilts.

Item	CON	SPV	*p*-Value
Caecal content, mg/g			
Acetate	2.7 ± 0.2	3.5 ± 0.3	0.04
Propionic acid	1.2 ± 0.1	1.4 ± 0.2	0.30
Butyric acid	0.6 ± 0.1	1.0 ± 0.2	0.07
Colonic content, mg/g			
Acetate	2.6 ± 0.2	3.6 ± 0.3	0.02
Propionic acid	1.0 ± 0.1	1.2 ± 0.1	0.15
Butyric acid	0.7 ± 0.1	1.2 ± 0.2	0.04

CON, gilts fed the basal diet; SPV, gilts fed basal diet with sweet potato vine. Values are mean ± SEM (n = 10).

**Table 5:** Tags and Operational Taxonomic Units (OTUs) between the treatments.

Item	CON	SPV
Total_tag	55,402 ± 2101	59,469 ± 2144
Taxon_Tag	52,187 ± 1876	56,509 ± 2026
Unique_Tag	3215 ± 303	2960 ± 182
OTU_num	1876 ± 155	1852 ± 109

CON, gilts fed the basal diet; SPV, gilts fed basal diet with sweet potato vine. Values are mean ± SD (n = 8).

**Table 6:** The effect of sweet potato vine treatment on the relative abundances of fifteen phyla (%, ≥0.01% of total abundances) in Meishan gilts.

Item	CON	SPV	*p*-Value
*Firmicutes*	45.21 (40.61; 50.33)	45.66 (41.20; 50.61)	0.88
*Bacteroidetes*	28.93 (25.28; 33.11)	29.15 (26.20; 32.44)	0.92
*Spirochaetes*	4.69 (2.87; 7.65)	4.37 (3.52; 5.41)	0.75
*Proteobacteria*	4.32 (2.38; 7.81)	3.91 (3.27; 4.66)	0.71
*Tenericutes*	1.78 (1.48; 2.15)	1.58 (1.29; 1.93)	0.32
*Actinobacteria*	1.20 (0.50; 2.90)	1.45 (0.75; 2.79)	0.69
*Verrucomicrobia*	1.03 (0.62; 1.72)	0.56 (0.44; 0.71)	0.02
*Acidobacteria*	0.42 (0.10; 1.70)	0.34 (0.14; 0.84)	0.78
*Gemmatimonadetes*	0.13 (0.03; 0.62)	0.07 (0.03; 0.17)	0.43
*Chloroflexi*	0.16 (0.04; 0.74)	0.18 (0.07; 0.44)	0.93
*Cyanobacteria*	0.22 (0.15; 0.32)	0.27 (0.19; 0.39)	0.33
*Fibrobacteres*	0.15 (0.09; 0.24)	0.11 (0.08; 0.15)	0.24
*Euryarchaeota*	0.09 (0.04; 0.14)	0.04 (0.02; 0.07)	0.09
*Lentisphaerae*	0.03 (0.02; 0.06)	0.01 (0.006; 0.03)	0.09
*Armatimonadetes*	0.04 (0.01; 0.13)	0.01 (0.004; 0.04)	0.16

Data were log-transformed before statistical analysis, and hence confidence limits are given in brackets instead of SEM values. Gilts were regarded as the experimental units, n = 8. CON, gilts fed the basal diet; SPV, gilts fed basal diet with sweet potato vine.

**Table 7:** The effect of sweet potato vine treatment on the relative abundances of eighteen genera (%, >0.3% of total abundances) in Meishan gilts.

Item	CON	SPV	*p*-Value
*Prevotella*	4.17 (3.23; 5.38)	5.54 (4.08; 7.52)	0.12
*Treponema*	4.60 (2.78; 7.63)	4.30 (3.46; 5.34)	0.76
*Lactobacillus*	1.43 (0.90; 2.26)	2.70 (1.88; 3.88)	0.02
*CF231*	2.52 (1.86; 3.41)	2.70 (1.96; 3.72)	0.71
*Oscillospira*	2.52 (2.14; 2.98)	2.26 (1.82; 2.80)	0.36
*Clostridium*	1.56 (1.22; 1.99)	1.58 (1.24; 2.00)	0.94
*Bacteroides*	1.00 (0.84; 1.19)	1.56 (1.22; 1.99)	0.01
*Ruminococcus*	1.56 (1.39; 1.75)	1.51 (1.25; 1.83)	0.78
*[Prevotella]*	0.94 (0.64; 1.39)	1.34 (0.98; 1.84)	0.12
*Roseburia*	0.73 (0.59; 0.92)	1.04 (0.78; 1.39)	0.04
*Streptococcus*	0.67 (0.52; 0.87)	0.78 (0.45; 1.38)	0.57
*Parabacteroides*	0.76 (0.58; 1.01)	0.89 (0.69; 1.15)	0.35
*Lachnospira*	0.30 (0.24; 0.38)	0.71 (0.48; 1.05)	< 0.01
*SMB53*	0.85 (0.69; 1.04)	0.61 (0.54; 0.70)	< 0.01
*Anaerovibrio*	0.35 (0.27; 0.46)	0.51 (0.34; 0.77)	0.10
*Coprococcus*	0.50 (0.39; 0.64)	0.59 (0.48; 0.72)	0.23
*Turicibacter*	0.66 (0.45; 0.97)	0.49 (0.38; 0.64)	0.14
*Epulopiscium*	0.36 (0.28; 0.48)	0.46 (0.32; 0.68)	0.25

Data were log-transformed before statistical analysis, and hence confidence limits are given in brackets instead of SEM values. Gilts were regarded as the experimental units, n = 8. CON, gilts feed the basal diet; SPV, gilts fed basal diet with sweet potato vine.

## References

[1] Zhang P., Cao M., Li J., Lin Y., Fang F., Che L., Feng B., Zhuo Y., Wang J., Wu D. (2019). Effect of sweet potato vine on the onset of puberty and follicle development in Chinese Meishan gilts. Animals.

[2] Geuking M.B., Köller Y., Rupp S., McCoy K.D. (2014). The interplay between the gut microbiota and the immune system. Gut Microbes.

[3] Duncan S.H., Louis P., Thomson J.M., Flint H.J. (2009). The role of pH in determining the species composition of the human colonic microbiota. Environ. Microbiol..

[4] Mueller S., Saunier K., Hanisch C., Norin E., Alm L., Midtvedt T., Cresci A., Silvi S., Orpianesi C., Verdenelli M.C. (2006). Differences in fecal microbiota in different european study populations in relation to age, Gender, and Country: A Cross-Sectional Study. Appl. Environ. Microbiol..

[5] De F.C., Cavalieri D., Di P.M., Ramazzotti M., Poullet J.B., Massart S., Collini S., Pieraccini G., Lionetti P. (2010). Impact of diet in shaping gut microbiota revealed by a comparative study in children from Europe and rural Africa. Proc. Natl. Acad. Sci. USA.

[6] Conlon M.A., Bird A.R. (2015). The impact of diet and lifestyle on gut microbiota and human health. Nutrients.

[7] Zhou P., Zhao Y., Zhang P., Li Y., Gui T., Wang J., Jin C., Che L., Li J., Xu S. (2017). Microbial mechanistic insight into the role of inulin in improving maternal health in a pregnant sow model. Front. Microbiol..

[8] Zou G.Y. (1991). Sweet potato and its vine leaves are worth to develop as food sources. Food Sci. (China).

[9] Zhang L.M., Wang Q.M., Wang Y.C.H. (2003). The Main Nutrient Components and Health Care Function of Sweet Potato. Rain Fed Crops.

[10] Chen H., Mao X., Che L., Yu B., He J., Yu J., Han G., Huang Z., Zheng P., Chen D. (2014). Impact of fiber types on gut microbiota, gut environment and gut function in fattening pigs. Anim. Feed Sci. Technol..

[11] Weitkunat K., Schumann S., Petzke K.J., Blaut M., Loh G., Klaus S. (2015). Effects of dietary inulin on bacterial growth, short-chain fatty acid production and hepatic lipid metabolism in gnotobiotic mice. J. Nutr. Biochem..

[12] Duncan S.H., Hold G.L., Barcenilla A., Stewart C.S., Flint H.J. (2002). Roseburia intestinalis sp. nov., a novel saccharolytic, butyrate-producing bacterium from human faeces. Int. J. Syst. Evol. Microbiol..

[13] Van d.A.P., Gérard P., Rabot S., Bruneau A., El Aidy S., Derrien M., Kleerebezem M., Zoetendal E.G., Smidt H., Verstraete W. (2011). Arabinoxylans and inulin differentially modulate the mucosal and luminal gut microbiota and mucin-degradation in humanized rats. Environ. Microbiol..

[14] AOAC (2000). Official Methods of Analysis.

[15] Rehfeldt C., Stabenow B., Pfuhl R., Block J., Nürnberg G., Otten W., Metges C.C., Kalbe C. (2012). Effects of limited and excess protein intakes of pregnant gilts on carcass quality and cellular properties of skeletal muscle and subcutaneous adipose tissue in fattening pigs. J. Anim. Sci..

[16] Wang D., Xu S., Lin Y., Fang Z., Che L., Xue B., Wu D. (2014). Recombinant porcine epidermal growth factorsecreting Lactococcus lactis promotes the growth performance of early-weaned piglets. BMC Vet. Res..

[17] Livak K.J., Schmittgen T.D. (2001). Analysis of relative gene expression data using real-time quantitative PCR and the 2^−ΔΔ*C*^_T_ Method. Methods.

[18] Holt J.P., Johnston L.J., Baidoo S.K., Shurson G.C. (2006). Effects of a high-fiber diet and frequent feeding on behavior, reproductive performance, and nutrient digestibility in gestating sows. J. Anim. Sci..

[19] Gall M.L., Warpechowski M., Jaguelinpeyraud Y., Noblet J. (2009). Influence of dietary fibre level and pelleting on the digestibility of energy and nutrients in growing pigs and adult sows. Animal.

[20] Wang C., Lin H., Wang Y., Zhang Y., Wang H., Wu Y. (2011). Effect of dietary fiber levels on appearance digestibility of nutrients. China Animal Husb. & Vet. Med..

[21] Peng B., Ga Y., Wang C., Wang Y. (2011). Effects of fresh alfalfa levels and alfalfa meal in the diet on the production performance of sows. Acta Pratacultureae Sin. (China).

[22] Yang Y.F. (2001). Effect of Dietary Fiber on Digestive Physiology and Production Performance of Pigs at Different Growth Stages. Ph.D. Thesis.

[23] Len N.T., Hong T.T., Ogle B., Lindberg J.E. (2009). Comparison of total tract digestibility, development of visceral organs and digestive tract of Mong cai and Yorkshire x Landrace piglets fed diets with different fibre sources. J. Anim. Physiol. Anim. Nutr..

[24] Rozeboom D.W., Pettigrew J.E., Moser R.L., Cornelius S.G., EL Kandelgy S.M. (1995). Body composition of gilts at puberty. J. Anim. Sci..

[25] Jonathan M.C., Van den Borne J.J.G.C., Van Wiechen P., Da Silva C.S., Schols H.A., Gruppen H. (2012). In vitro fermentation of 12 dietary fibres by faecal inoculum from pigs and humans. Food Chem..

[26] Lin B., Gong J., Wang Q., Cui S., Yu H., Huang B. (2011). In-vitro assessment of the effects of dietary fibers on microbial fermentation and communities from large intestinal digesta of pigs. Food Hydrocoll..

[27] Salvador V., Cherbut C., Barry J.L., Bertrand D., Bonnet C., Delort-Laval J. (1993). Sugar composition of dietary fibre and short-chain fatty acid production during in vitro fermentation by human bacteria. Br. J. Nutr..

[28] Fushimi T., Suruga K., Oshima Y., Fukiharu M., Tsukamoto Y., Goda T. (2006). Dietary acetic acid reduces serum cholesterol and triacylglycerols in rats fed a cholesterol-rich diet. Br. J. Nutr..

[29] Queenan K.M., Stewart M.L., Smith K.N., Thomas W., Fulcher R.G., Slavin J.L. (2007). Concentrated oat beta-glucan, a fermentable fiber, lowers serum cholesterol in hypercholesterolemic adults in a randomized controlled trial. Nutr. J..

[30] Van Hoek M.J.A., Merks R.M.H. (2012). Redox balance is key to explaining full vs. partial switching to low-yield metabolism. BMC Syst. Biol..

[31] Fasano A. (2011). Zonulin and its regulation of intestinal barrier function: The biological door to inflammation, autoimmunity, and cancer. Physiol. Rev..

[32] Kelly C.J., Zheng L., Campbell E.L., Saeedi B., Scholz C.C., Bayless A.J., Wilson K.E., Glover L.E., Kominsky D.J., Magnuson A. (2015). Crosstalk between microbiota-derived short-chain fatty acids and intestinal epithelial HIF augments tissue barrier function. Cell Host Microbe.

[33] Akira S., Hirano T., Taga T., Kishimoto T. (1990). Biology of multifunctional cytokines: IL 6 and related molecules (IL 1 and TNF). FASEB J..

[34] Ley R., Turnbaugh P., Klein S., Gordon J.I. (2006). Human gut microbes associated with obesity. Nature.

[35] Tamanai-Shacoori Z., Smida I., Bousarghin L., Loreal O., Meuric V., Fong S.B., Bonnaure-Mallet M., Jolivet-Gougeon A. (2017). *Roseburia* spp.: A marker of health?. Future Microbiol..

[36] De Preter V., Machiels K., Joossens M., Arijs I., Matthys C., Vermeire S., Rutgeerts P., Verbeke K. (2015). Faecal metabolite profiling identifies medium-chain fatty acids as discriminating compounds in IBD. Gut.

[37] Weber F.H., Canaleparola E. (1984). Pectinolytic enzymes of oral spirochetes from humans. Appl. Environ. Microbiol..

[38] Ramirezfarias C., Slezak K., Fuller Z., Duncan A., Holtrop G., Louis P. (2008). Effect of inulin on the human gut microbiota: Stimulation of *Bifidobacterium adolescentis* and *Faecalibacterium prausnitzii*. Br. J. Nutr..

[39] Resta-Lenert S., Barrett K.E. (2003). Live probiotics protect intestinal epithelial cells from the effects of infection with enteroinvasive Escherichia coli (EIEC). Gut.

[40] Wang B., Li J., Li Q., Li Y., Li N., Li J. (2007). Effect of *Lactobacillus* in the intestinal barrier function on enteropathogenic Escherichia coli infected mice. Parenter. Eenter. Nutr. (China).

[41] Servin A.L., Coconnier M.H. (2003). Adhesion of probiotic strains to the intestinal mucosa and interaction with pathogens. Best Pract. Res. Clin. Gastroenterol..

[42] Doleyres Y., Beck P., Vollenweider S., Lacroix C. (2005). Production of 3 hydroxypropionaldehyde using a two-step process with *Lactobacillus reuteri*. Appl. Microbiol. Biotechnol..

